# Supplementary material for: HIVprotI: an integrated web based platform for prediction and design of HIV proteins inhibitors
Source: J Cheminform. 2018 Mar 9;10:12. doi: 10.1186/s13321-018-0266-y (PMC5845081; doi:10.1186/s13321-018-0266-y)
Supplement: Supplementary file 2 — Additional file 2. Source code of HIVProtI web server. [file 13321_2018_266_MOESM2_ESM.zip › HIVprotI_Source-code/hivprotI/weka-3-6-6/doc/weka/core/converters/package-summary.html]

weka.core.converters


---


|  |  |  |
| --- | --- | --- |
| **Overview** \| **Package** \| Class \| **Tree** \| **Deprecated** \| **Index** \| **Help** \| **Weka's home** | |  |
| **PREV PACKAGE**   **NEXT PACKAGE** | **FRAMES**    **NO FRAMES**     **All Classes** |  |


---

## Package weka.core.converters

| **Interface Summary** | |
| --- | --- |
| **BatchConverter** | Marker interface for a loader/saver that can retrieve instances in batch mode |
| **DatabaseConverter** | Marker interface for a loader/saver that uses a database |
| **FileSourcedConverter** | Interface to a loader/saver that loads/saves from a file source. |
| **IncrementalConverter** | Marker interface for a loader/saver that can retrieve instances incrementally |
| **Loader** | Interface to something that can load Instances from an input source in some format. |
| **Saver** | Interface to something that can save Instances to an output destination in some format. |
| **URLSourcedLoader** | Interface to a loader that can load from a http url |

| **Class Summary** | |
| --- | --- |
| **AbstractFileLoader** | Abstract superclass for all file loaders. |
| **AbstractFileSaver** | Abstract class for Savers that save to a file Valid options are: -i input arff file   The input filw in arff format. |
| **AbstractLoader** | Abstract class gives default implementation of setSource methods. |
| **AbstractSaver** | Abstract class for Saver |
| **ArffLoader** | Reads a source that is in arff (attribute relation file format) format. |
| **ArffLoader.ArffReader** | Reads data from an ARFF file, either in incremental or batch mode. |
| **ArffSaver** | Writes to a destination in arff text format. |
| **C45Loader** | Reads a file that is C45 format. |
| **C45Saver** | Writes to a destination that is in the format used by the C4.5 algorithm. |
| **ConverterUtils** | Utility routines for the converter package. |
| **ConverterUtils.DataSink** | Helper class for saving data to files. |
| **ConverterUtils.DataSource** | Helper class for loading data from files and URLs. |
| **CSVLoader** | Reads a source that is in comma separated or tab separated format. |
| **CSVSaver** | Writes to a destination that is in csv format   Valid options are: |
| **DatabaseConnection** | Connects to a database. |
| **DatabaseLoader** | Reads Instances from a Database. |
| **DatabaseSaver** | Writes to a database (tested with MySQL, InstantDB, HSQLDB). |
| **LibSVMLoader** | Reads a source that is in libsvm format.    For more information about libsvm see:    http://www.csie.ntu.edu.tw/~cjlin/libsvm/ |
| **LibSVMSaver** | Writes to a destination that is in libsvm format.    For more information about libsvm see:    http://www.csie.ntu.edu.tw/~cjlin/libsvm/   Valid options are: |
| **SerializedInstancesLoader** | Reads a source that contains serialized Instances. |
| **SerializedInstancesSaver** | Serializes the instances to a file with extension bsi. |
| **SVMLightLoader** | Reads a source that is in svm light format.    For more information about svm light see:    http://svmlight.joachims.org/ |
| **SVMLightSaver** | Writes to a destination that is in svm light format.    For more information about svm light see:    http://svmlight.joachims.org/   Valid options are: |
| **TextDirectoryLoader** | Loads all text files in a directory and uses the subdirectory names as class labels. |
| **XRFFLoader** | Reads a source that is in the XML version of the ARFF format. |
| **XRFFSaver** | Writes to a destination that is in the XML version of the ARFF format. |

---


|  |  |  |
| --- | --- | --- |
| **Overview** \| **Package** \| Class \| **Tree** \| **Deprecated** \| **Index** \| **Help** \| **Weka's home** | |  |
| **PREV PACKAGE**   **NEXT PACKAGE** | **FRAMES**    **NO FRAMES**     **All Classes** |  |


---
